# Evaluating the Impact of Hyperbaric Oxygen Therapy and Neurofeedback on Mild Traumatic Brain Injury: A Case Report

**DOI:** 10.7759/cureus.77228

**Published:** 2025-01-10

**Authors:** Tami Peterson, JeAnnah Rose AbouAssaly, Elizabeth Terry, Sheila Burgin, Robert Sherwin, Frederick Strale

**Affiliations:** 1 Hyperbaric Oxygen Therapy, The Oxford Center, Brighton, USA; 2 Neurofeedback, The Oxford Center, Brighton, USA; 3 Medical Services, The Oxford Center, Brighton, USA; 4 Hyperbaric Oxygen Therapy, Wayne State University School of Medicine, Detroit, USA; 5 Biostatistics, The Oxford Center, Brighton, USA

**Keywords:** hyperbaric oxygen therapy and neurofeedback therapy, hyperbaric oxygen therapy (hbot), mild tbi, mild traumatic brain injury (mtbi), neurofeedback therapy, post traumatic brain injury

## Abstract

A 38-year-old male patient sustained a concussion in May 2017 while playing hockey. Despite initially following standard treatments and rest, he continued to experience post-concussive symptoms. Approximately two weeks later, he sought alternative therapy at The Oxford Center in Brighton, Michigan, where he was introduced to hyperbaric oxygen therapy (HBOT). Although a 40-session protocol was recommended, he ended treatment after 11 sessions, noting subjective improvements but later recognizing that discontinuing early may have hindered full recovery.

Over time, the patient’s medical history, encompassing ocular migraines, chronic and adrenal fatigue, and recurrent infections, contributed to ongoing stressors. Seasonal variations and occupational demands further exacerbated his symptoms. In July 2018, a second head injury occurred during a jet-ski accident, leading to more pronounced cognitive and functional impairments. A renewed course of 40 HBOT sessions, followed by an additional 40, produced substantial improvement and subsequent neurofeedback interventions further enhanced cognitive function, emotional regulation, and overall quality of life. By early 2019, after roughly 100 HBOT sessions, the patient reported a near-complete return to baseline. He continued periodic HBOT for maintenance, describing it as a “breath of life.”

From 2018 to mid-2024, successive neurofeedback sessions, guided by quantitative electroencephalography (qEEG) assessments, yielded marked gains in decision-making, attentional focus, and mental flexibility. The patient also employed intravenous micronutrient therapies, particularly high-dose vitamin C, to bolster immune resilience following pneumonia. Integrating HBOT, neurofeedback, and targeted physiological support, this comprehensive approach underscores the potential for synergistic benefits in recurrent mild traumatic brain injury (mTBI).

The Neurobehavioral Symptoms Inventory (NSI) data reveal a statistically significant reduction in overall neurobehavioral symptoms from 2018 to 2024 (z = -2.754, p = .006, d = 0.692), with prominent declines in anxiety, poor concentration, and depressed mood, as well as smaller but meaningful improvements in dizziness, balance, coordination, decision-making, slowed thinking, and fatigue. Some symptoms - such as headaches, nausea, and sensitivity to noise - remained unchanged, indicating areas for continued monitoring and targeted strategies. These results suggest the beneficial effects of interventions or changes implemented during the six-year span, highlighting the potential for sustained improvement with ongoing support.

The patient remains engaged in both HBOT and neurofeedback, reporting sustained improvements in cognition, emotional balance, and stress management. This case highlights the importance of adhering to recommended HBOT protocols, using neurofeedback to address persistent neurocognitive deficits, and incorporating ongoing evaluations to refine therapeutic strategies. These findings suggest that a multifaceted, long-term treatment plan may optimize recovery in individuals with recurrent mTBI, offering insights for clinicians seeking integrative methods to promote durable neurological and functional outcomes.

## Introduction

Thousands of individuals in the United States daily experience concussions due to incidents such as vehicular accidents, falls, and participation in sports. The Centers for Disease Control and Prevention (CDC) approximates that annually, as many as 3.8 million concussions, also referred to as mild traumatic brain injury (mTBI), are related to sports and recreational activities within the U.S. [1].

Post-concussion syndrome (PCS) refers to persisting cognitive, emotional, and behavioral problems that a subset of patients continues to report after a mild mTBI. It is a disorder impacting roughly 10 to 15 percent of people who have experienced a concussion. This syndrome is marked by persistent symptoms that can last for weeks, months, or even years after the initial trauma. These symptoms can manifest as headaches, dizziness, and cognitive or emotional disturbances. While most concussions typically resolve within a short period, PCS is distinguished by a prolonged duration of symptoms. It is crucial for individuals experiencing ongoing symptoms after a concussion to seek a medical assessment and the necessary care [1].

PCS garners significant attention from the professional community and the public. However, there is a widespread lack of clarity surrounding its origin, causes, diagnostic methods, symptom manifestations, factors that extend its duration, and treatment approaches [2]. Dhandapani et al. systematically reviewed randomized controlled trials to identify the clinical shortcomings in managing PCS and pinpoint factors that can prevent long-term neurobehavioral issues [3]. The studies reviewed indicated that effective PCS management can significantly enhance quality of life, reduce the financial strain on the healthcare system, and potentially mitigate long-term complications like depression and anxiety. Beginning treatment as soon as possible is crucial, which includes educating patients about the expected progression after mTBI and identifying those at risk for enduring symptoms. Given that PCS encompasses a range of symptoms, it’s essential to identify and individually treat the primary symptom. A multidisciplinary team approach is recommended for optimal results, incorporating interdisciplinary treatments [3].

Neurofeedback treatments

Psychophysiological biofeedback (BFB) protocols such as quantitative electroencephalography (qEEG) and neurofeedback offer a crucial advantage in the post-concussion treatment of mTBI by providing sensitive, objective physiological measures and treatments. These measures can offer data beyond subjective symptom reports, as there are no universally accepted biomarkers for concussion. The Institute of Medicine has noted changes in various neurophysiological parameters post-concussion. Neurofeedback data can help objectify athlete symptoms, identify dysregulated systems, and pinpoint targets for neurofeedback and psychotherapeutic interventions to teach self-regulation [2].

qEEG neurofeedback serves as a potent instrument in the examination of PCS. It provides a factual, unbiased, and non-disruptive approach to evaluation. Studies have demonstrated its high accuracy in identifying and distinguishing the various neurophysiological patterns of brain dysfunction linked to minor TBI and PCS [4,5].

Using qEEG, clinicians can gain deeper insights into the underlying neurophysiological factors contributing to PCS symptoms. This functional information enables the development of more precise and targeted treatment options, enhancing patient care. Additionally, in medical and legal contexts, qEEG provides robust empirical evidence of functional abnormalities that correspond with the symptoms of post-concussion syndrome resulting from traumatic brain injuries. This evidence can be crucial in substantiating claims and supporting case arguments [5].

Hyperbaric oxygen therapy (HBOT) treatments with mild TBI

Harch et al. found that 63 participants with 150 kPa HBOT, approximately 1.48 absolute atmospheres (ATA), significantly improved on various measures, including cognition, mood, PCS, sleep, and quality of life [6]. Harch conducted a systematic review and dosage analysis with HBOT efficacy in mild traumatic brain injury persistent post-concussion syndrome. In numerous randomized and controlled studies, Harch found that HBOT at 1.5 ATA oxygen has shown significant symptomatic and cognitive improvements in patients with mTBI and PCS. Both positive and negative outcomes were observed at lower and higher doses of oxygen and pressure. Increased pressure within a specific range appears more impactful than increased oxygen is effective over a wide range [7]. The improvements were more pronounced in patients who also had post-traumatic stress disorder. Despite the small sample sizes, the 1.5 ATA HBOT studies meet the Level 1 criteria of the Centre for Evidence-Based Medicine and receive a Class A Recommendation from the American Society of Plastic Surgeons for treating mTBI and persistent PCS [7].

Heslot et al. systematically reviewed five randomized controlled trials on the efficacy of HBOT with PCS and found contradictory results (two positive and three negative) [8]. They mentioned Harch et al. [6] and another study including 56 patients [9], that HBOT improved cognitive functions and quality of life. However, three other randomized sham-controlled trials [10-12] did not find any cognitive improvement after HBOT in a large sample of participants suffering from PCS after mTBI.

The outcomes of HBOT in reducing mTBI symptoms can vary due to several factors such as patient individual differences, timing of treatment, dosage and protocol variations, placebo effect, comorbid conditions, and measurement methods. Also, the potential risks of HBOT include barotrauma, oxygen toxicity, and fire hazard. Potential contraindications include: untreated pneumothorax, severe chronic obstructive pulmonary disease (COPD) or asthma, potential ear surgery or injury, claustrophobia, and certain chemotherapy drugs. It’s essential for patients to have a comprehensive medical evaluation before beginning HBOT to confirm its safety for them.

Neurofeedback treatments with mild TBI

Cognitive impairment is a prevalent and disabling outcome of mTBI, affecting individuals' ability to return to work, reducing quality of life, and imposing significant socioeconomic and healthcare burdens. Common cognitive deficits include memory, attention, and information processing impairments, which can also affect higher-level functions like executive function. Preserving essential cognitive functions prevents or delays posttraumatic cognitive impairment [13]. Cognitive rehabilitation is the most commonly prescribed treatment for post-TBI cognitive impairment, but evidence from meta-analyses of randomized controlled trials (RCTs) has been mixed. Pharmacotherapy, such as methylphenidate, has been used to speed up cognitive recovery, though systematic reviews have not consistently shown its effectiveness. Therefore, alternative approaches are needed to reduce the risk of cognitive impairment after TBI [13].

May et al. conducted a literature review of 22 published papers that measured symptom improvement, neuropsychological testing, and changes in subjects’ qEEG [14]. All studies demonstrated positive findings in that neurofeedback led to improvement in measures of impairment, whether subjective, objective, or both. They concluded that neurofeedback is a promising therapy in the treatment of TBI. Clinicians can advise that some patients report improvement in a wide range of neuropsychiatric symptoms [14].

Munivenkatappa et al. conducted a study involving two patients, averaging 15 years of age, who had sustained moderate brain injuries, with computer tomography scans indicating potential diffuse axonal injury. Both patients underwent 20 neurofeedback sessions, lasting 40 minutes per session, three times a week over two months. The neurofeedback protocol targeted theta (4-7 Hz) and alpha (8-12 Hz) wave frequencies. Pre- and post-treatment neuropsychological assessments, including the Rivermead Concussion Symptoms Scale and the NIMHANS Neuropsychology Battery, revealed improvements in mental speed, working memory, and visual memory retrieval [15,16].

Rostami et al. conducted a randomized controlled trial with 13 patients with moderate TBI, aged 15 to 60, who received neurofeedback training. Eight patients in the intervention group participated in 20 sessions over four weeks, while the control group, consisting of five patients, began their sessions during the fifth to eighth week of the study. The neurofeedback protocols involved beta and alpha coherence methods, with participants keeping their eyes open during 50-minute sessions. Electrodes were placed at the FP1-T3 and Cz-Oz regions. Despite the intervention, assessments using the Wechsler Memory Scale (WMS-IV) and the Continuous Attention Test (DAUF test) did not show statistically significant improvements in short-term memory, long-term attention, or concentration performance [15,17].

Arroyo-Ferrer et al. compared neurofeedback to traditional cognitive rehabilitation methods in a study involving a 20-year-old brain injury patient and three healthy controls [18]. Participants underwent neurofeedback training focused on inhibiting the theta band, each receiving eight 45-minute sessions over two weeks. Visual feedback was provided using three different visual scenarios. The study extended over six weeks, with the patient taking a two-week rest before engaging in conventional rehabilitation during the final two weeks. Improvements in attention, measured by the Brief Test of Attention (BTA), were observed following both neurofeedback and traditional methods. Short-term memory showed improvement after conventional rehabilitation, as measured by the Rey-Osterrieth Complex Figure (ROCF) and the TAVEC-Verbal Learning Test Espana Complutense, while delayed memory improvement was noted following neurofeedback training [15,18].

Combined HBOT and neurofeedback treatments for TBI

Few studies exist looking at the combined efficacy of HBOT and neurofeedback treatments in reducing mTBI.

White et al. [19] describe a 2014 case of a 26-year-old male with severe TBI from a car accident, involving injuries to the left temporal and frontal areas. After a left-side craniotomy and 26 days in a coma, he spent eight months receiving rehabilitation therapies (physical, speech, and occupational) but still faced significant impairments (speech, mobility, spasticity, cognition, and epilepsy). His parents then sought hyperbaric oxygen therapy (HBOT), completing 165 sessions before adding neurofeedback therapy in March 2019. This combined treatment led to notable improvements in brain function, including better memory, personality, language, and executive function, along with fewer seizures. Given the often-limited recovery outlook for severe TBI, the authors stress the need for more long-term, effective treatments. Their study appears to be the first to explore combining HBOT and neurofeedback for TBI, indicating a promising therapeutic direction that warrants further research.

Peterson et al. [20] extended the research of White et al. [19], finding, following 195 neurofeedback sessions and more than 300 HBOT treatments, additional improvements in cognitive and emotional well-being, as well as in daily living activities such as feeding, toileting, grooming, and communication. A post-treatment qEEG conducted in June 2024 revealed moderate to large effects on the brain’s average frequency band parameters (g = .612) and small to moderate effects across 19 scalp electrode placements (uV^2 g = .339; Hz g = .333), indicating considerable progress over a 31-month treatment period [20].

According to objective measures - including the Disability Rating Scale (DRS) and the Glasgow Outcome Scale Extended (GOSE) - the patient’s case showed significant gains in feeding (p = .046), toileting (p = .046), grooming (p = .046), and communication abilities (p = .046). Based on qEEG effect sizes, along with DRS and GOSE results from pretest (2021) to posttest (2024), she has demonstrated marked improvements in both brain recovery and overall quality of life.

The synergy between HBOT and neurofeedback could be a viable and effective treatment option for TBI, warranting further research and clinical application. Given this scarcity of relevant research, and building upon the foundational research studies mentioned, we present a comprehensive case report detailing the mTBI and subsequent therapeutic recovery trajectory of a 38-year-old male. This report aims to elucidate the clinical manifestations, diagnostic evaluations, and therapeutic interventions employed, thereby contributing to the broader understanding of mTBI management and recovery trajectories with combined HBOT and neurofeedback treatments.

## Case presentation

Initial injury (May 2017)

In May 2017, a 38-year-old male patient sustained a concussion while playing hockey. Although he initially followed standard treatments and adhered to a period of rest, his post-concussive symptoms persisted. Approximately two weeks after the injury, he sought alternative interventions, recalling radio advertisements for The Oxford Center in Brighton, Michigan. Upon contacting the center, he consulted with our medical providers, who explained the potential benefits of HBOT in promoting neuro-regeneration and mitigating lingering post-concussive symptoms.

He subsequently initiated HBOT sessions and observed measurable improvements after the first treatments. Although a 40-session protocol had been recommended, he chose to discontinue therapy after 11 sessions, reporting a subjective resolution of symptoms. In hindsight, the patient acknowledged that ending treatment prematurely may have left his brain only partially healed, thereby increasing susceptibility to further complications.

In addition to his concussive injuries, the patient’s medical history encompassed ocular migraines, chronic and adrenal fatigue, recurrent infections, and episodes of pneumonia, from which he has recovered without complications. He continues to experience fatigue and recurrent staphylococcal infections, underscoring a familial predisposition to autoimmune disorders. Seasonal shifts and heightened stress, particularly from running a landscaping business, exacerbate his symptoms, highlighting the impact of environmental and occupational factors on overall immune resilience.

Second injury (July 2018)

In July 2018, the patient was involved in a jet-ski accident in Lake Huron. While navigating large waves, the patient misjudged the height of a swell and experienced a forceful freefall of approximately 10 feet. Upon landing, the patient sustained significant whiplash and suspected a head injury but continued jet-skiing, potentially exacerbating the trauma. Despite a growing awareness of possible cerebral injury, the patient returned home several days later, driving approximately four hours. Shortly thereafter, the patient developed pronounced brain fog, reduced appetite, lethargy, and a marked inability to perform routine tasks, symptoms indicative of a traumatic brain injury.

The patient contacted Oxford again and was assessed by a clinician at the Brighton facility. Given the severity of symptoms, a regimen of 40 HBOT sessions was recommended. Throughout these treatments, the patient experienced a progressive improvement in basic functioning. During the initial two-week period, the patient was unable to drive independently and spent most of each day confined to bed, except when attending HBOT sessions. Minimal energy was available for daily activities, and the patient reported forcing food intake purely out of physiological necessity.

Subsequent therapeutic interventions

Following the initial 40 HBOT sessions for the second TBI, the patient elected to complete an additional 40 treatments, totaling 80 sessions. During the latter part of that year, the patient also underwent neurofeedback treatments to address lingering cognitive and emotional disturbances. The patient received 20 neurofeedback sessions, coinciding with continued HBOT, resulting in progressive improvement in neurological function. By early 2019, after approximately 100 HBOT sessions, the patient reported feeling a near-complete return to baseline function. The patient also consulted with an in-house physician at Oxford, who guided neurotransmitter function and genetic factors that could influence recovery. This additional insight allowed the patient to target underlying issues predating the mTBIs.

Over the ensuing years, the patient continued to utilize HBOT regularly, becoming a “frequent flyer” at Oxford. The patient reported that repeated sessions helped mitigate high-stress levels associated with work and assisted in overall neurological maintenance. According to the patient, HBOT served as a “breath of life,” promoting both physical and psychological well-being.

During neurofeedback treatments conducted in late 2018 and again in early to mid-2024, the patient reported noteworthy progress across several cognitive and emotional functioning domains. The patient experienced enhanced decision-making capacity, evidenced by more efficient and consistent judgments during daily activities and social interactions. The patient also noted a marked improvement in attentional focus, enabling more sustained engagement with tasks or extended conversations without distraction.

The patient observed reduced mental fatigue, reporting fewer instances of feeling mentally “drained” after minimal cognitive exertion and increased mental clarity and processing speed. The previously reported slowness in reasoning, manifested as difficulty in processing information or formulating decisions, diminished substantially, allowing the patient to respond more promptly to complex or changing situations.

Regarding emotional regulation, the patient experienced fewer mood fluctuations and reported a decreased susceptibility to irritability, suggesting improved self-control in emotionally charged settings. Additionally, the patient described an enhanced capacity for cognitive flexibility, marked by a smoother transition between diverse tasks or topics and an increased ability to adapt to new or rapidly evolving circumstances. These collective improvements suggest that neurofeedback training may have played a significant role in restoring or augmenting this patient's higher-order cognitive and affective processes, resulting in enhanced daily functioning and overall quality of life.

In December 2022, the patient began receiving Myer cocktail infusions, an intravenous therapy typically comprising a blend of vitamins, minerals, and other micronutrients intended to bolster immune function and metabolic processes. Although he did not perceive dramatic or immediate shifts in day-to-day well-being, the patient expressed confidence in these treatments' internal, longer-term health benefits. Notably, he experienced marked improvements following high-dose vitamin C infusions, particularly after a pneumonia episode, which he credits with expediting his recovery and alleviating respiratory symptoms. This observation aligns with emerging evidence that vitamin C may exert immunomodulatory and antioxidant effects, potentially enhancing resilience during physiological stress or illness.

Follow-up and ongoing care

Neurofeedback and qEEG Reevaluation (Late Winter 2024)

In February-March 2024, the patient noted ongoing emotional dysregulation, including increased irritability that family members observed. The patient underwent a new qEEG assessment, recognizing the potential for unresolved neural dysregulation. The mapping revealed residual areas of dysregulated activity that warranted intervention. Consequently, the patient resumed neurofeedback training, completing 20 sessions. A subsequent qEEG demonstrated significant improvements, although specific brain regions required further retraining. At the time of this report, the patient is midway through a second set of 20 neurofeedback sessions, targeting the remaining areas of dysregulation. The patient reports feeling markedly better and more emotionally stable.

Ongoing HBOT Maintenance

Throughout this period, the patient continued to utilize HBOT as needed to address job-related stress and to maintain overall cognitive function. The patient attributes these ongoing sessions to sustaining a sense of neurological resilience and reports frequent, subjective energy, mood, and mental clarity improvements. Please see Table 1 below for patient neurobehavioral progress and inferential results.

**Table 1:** Patient Neurobehavioral Symptoms Scale (NSI) Outcomes 0 = None – Rarely if ever-present; not a problem at all 1 = Mild – Occasionally present, but it does not disrupt my activities; I can usually continue what I’m doing; doesn’t really concern me. 2 = Moderate – Often present, occasionally disrupts my activities; I can usually continue what I’m doing with some effort; I feel somewhat concerned. 3 = Severe – Frequently present and disrupts activities; I can only do things that are fairly simple or take little effort; I feel I need help. 4 = Very Severe – Almost always present and I have been unable to perform at work, school or home due to this problem; I probably cannot function without help.

Neurobehavioral Symptoms Inventory (NSI) Scale	Pretest (2018)	Posttest (2024)	Improvement	Overall Outcomes
Feeling Dizzy	1	0	1 Point Improvement	
Loss of balance	1	0	1 Point Improvement	
Poor coordination, clumsy	2	1	1 Point Improvement	
Headaches	0	0	N/A	
Nausea	0	0	N/A	
Vision problems, blurring, trouble seeing	0	0	N/A	
Sensitivity to light	0	0	N/A	
Hearing difficulty	0	0	N/A	
Sensitivity to noise	0	0	N/A	Wilcoxon Signed Rank z =-2.754, p=.006, d=0.692, 95%CI for d = 0.219-1.152
Numbness or tingling on parts of my body	0	0	N/A	
Change in taste and/or smell	0	0	N/A	Statistically Significant Gains in Overall Neurobehavioral Symptoms
Loss of appetite or increased appetite	0	0	N/A	
Poor concentration, can’t pay attention, easily distracted	3	1	2 Point Improvement	
Forgetfulness, can’t remember things	1	1	No Improvement	
Difficulty making decisions	2	1	1 Point Improvement	
Slowed thinking, difficulty getting organized, can’t finish things	1	0	1 Point Improvement	
Fatigue, loss of energy, getting tired easily	2	1	1 Point Improvement	
Difficulty falling or staying asleep	0	0	N/A	
Feeling anxious or tense	4	1	3 Point Improvement	
Feeling depressed or sad	2	0	2 Point Improvement	
Irritability, easily annoyed	1	1	No Improvement	
Poor frustration tolerance, feeling easily overwhelmed by things	1	1	No Improvement	

Method of intervention: neurofeedback

1. BrainMaster Discovery 24-Channel EEG Amplifier:* *The BrainMaster Discovery (BrainMaster Technologies, Bedford, OH, USA) is a compact system that measures and provides feedback on the brain’s electrical signals (EEG) in real-time. It records at a high resolution (24-bit) and a fast sampling rate (1,024 samples/second), covering a broad range of frequencies. With 24 channels (22 in a standard electrode cap and 2 additional reference channels), it can capture detailed information about brain activity. Because it’s powered via USB, it’s easy to use in clinics or even at home - there’s no need for batteries. This makes it both safe and convenient for neurofeedback sessions, where immediate feedback on brain activity helps guide changes in brain function [21].

2. WaveGuard Connect-19 EEG Cap:* *The WaveGuard Connect-19 (Advanced NeuroTechnology, Enschede, Netherlands) is an EEG cap designed for comfort and efficiency. It uses soft silicone electrode cups and durable tin electrodes that pick up high-quality brain signals. Its electrodes are pre-positioned according to a standard layout (the 10/20 system), so fitting the cap and applying gel takes less than 10 minutes. This reduces preparation time and makes it easier for both clinicians and patients. The cap’s wiring and connectors are designed to be reliable and straightforward, so it’s compatible with many EEG amplifiers in both routine checkups and neurofeedback sessions [22].

3. WinEEG Software*: *WinEEG (Mitsar Co. Ltd., St. Petersburg, Russia) is a user-friendly program for reviewing and analyzing EEG (brainwave) data after it’s been recorded. It supports multiple file formats (including EDF and EDF+) and can handle up to 256 channels of data. WinEEG helps remove artifacts (like blinks or muscle movements) through advanced methods such as ICA (Independent Component Analysis), making the data cleaner and more accurate. It also offers tools to visualize and compare data, including 3D mapping (LORETA), which shows where in the brain certain activities might be happening. Clinicians can easily export results to other programs for further review, track patient progress over time, and create reports that show changes in brain activity and function [23].

The protocols described here (including detailed EEG recording, comfortable and efficient equipment, and advanced yet accessible software) are selected because they ensure high-quality data. Accurate and detailed EEG signals help therapists tailor training protocols to each patient’s needs. They also offer comfort and convenience. Tools like the WaveGuard cap reduce setup time and discomfort, allowing the patient to focus on the training itself. These protocols also enable advanced analysis and feedback. WinEEG provides sophisticated ways to analyze and visualize brain activity, making it easier to track improvements and customize treatment. Together, these components support effective neurofeedback sessions, helping patients with mTBI potentially improve cognitive function, emotional balance, and overall quality of life [21-23].

Method of intervention: HBOT

From May 2018 through December 2024, The Oxford Center in Brighton, United States, employed a Class B monoplace hyperbaric chamber (Sechrist 3300H, Sechrist Industries, Inc., Anaheim, CA, USA) to administer hyperbaric oxygen therapy (HBOT) to the patient diagnosed with mTBI. The chamber’s standard operating procedure involved filling the interior with medical-grade oxygen, maintaining an oxygen concentration of approximately 100%. Treatment pressures were systematically elevated to 1.5 and 2.0 atmospheres absolute (ATA), with pressurization adjusted at a controlled rate of 1-2 psi/min. The frequency of these sessions reached up to five per week, depending on the patient’s tolerance and clinical response. Throughout each treatment, trained hyperbaric technicians vigilantly observed the patient for signs of adverse reactions, such as discomfort or oxygen toxicity. At the end of each session, the chamber was slowly returned to 1.0 ATA (sea-level atmospheric pressure) via a controlled depressurization rate of approximately 1-2 psi/min.

Before each therapy session, a Certified Hyperbaric Technician (CHT) conducted a thorough pre-treatment evaluation. This screening entailed thoroughly reviewing the patient’s medical history, establishing specific therapeutic objectives, and discussing potential risks, benefits, and contraindications. Particular attention was paid to ensuring the patient understood how to equalize ear pressure to avert barotrauma, employing a technique analogous to that used when flying on a commercial aircraft.

As a safety precaution, the patient was dressed in hospital-issued scrubs and advised to remove all metallic or electronic items, such as jewelry, eyeglasses, dentures, or contact lenses, to reduce the risk of damage or combustion in the elevated oxygen environment. Once preparations were complete, the patient was gently placed inside the monoplace chamber, which was sealed and pressurized. Communication was maintained throughout the procedure via an intercom system, allowing immediate feedback and monitoring for any emergent discomfort.

HBOT sessions were conducted from 2 to 5 times per week and lasted 120 minutes, consistent with the treatment protocol and the patient’s clinical status. After each session's completion, the chamber was gradually depressurized to standard atmospheric pressure. This deliberate transition was designed to minimize the risk of decompression-related complications. The patient was then escorted out of the chamber, rehydrated, and instructed to rest before resuming everyday activities. The patient was counseled on maintaining adequate fluid intake and limiting strenuous exertion for several hours post-treatment to facilitate optimal recovery and physiological adaptation. The patient did not encounter any adverse effects attributable to HBOT.

## Discussion

The results of the Neurobehavioral Symptom Inventory (NSI) indicate a statistically significant reduction in overall neurobehavioral symptoms from the pretest in 2018 to the posttest in 2024, as evidenced by the Wilcoxon Signed Rank test (z = -2.754, p = .006) and a medium effect size (d = 0.692, 95% CI = 0.219-1.152). Several symptoms demonstrated notable individual improvements. For instance, “feeling anxious or tense” decreased by three points (from 4 to 1), while “poor concentration” and “feeling depressed or sad” each declined by two points. These changes suggest meaningful clinical progress in areas most disruptive to daily functioning, such as mood and cognition.

Milder one-point reductions were observed in “feeling dizzy,” “loss of balance,” “poor coordination,” “difficulty making decisions,” “slowed thinking,” and “fatigue.” These improvements likely contributed to the overall positive outcome measured by the inventory. Importantly, some symptoms such as “headaches,” “nausea,” or “sensitivity to noise” did not change from baseline, reflecting either the absence of these difficulties or their relative stability over time.

The pattern of results underscores a significant improvement in key neurobehavioral symptoms, aligning with the moderate-to-large effect size estimate. Although not all symptoms improved, the data suggest that interventions or changes made during the six-year interval were beneficial for many of the areas assessed. Continued monitoring and targeted strategies may help sustain and further enhance these gains, especially for those remaining unchanged symptoms.

This case underscores the multifaceted nature of repeat TBI management, highlighting how a structured combination of HBOT and neurofeedback can address both structural and functional aspects of brain recovery. Although the patient observed notable progress after the initial HBOT sessions, prematurely discontinuing treatment following the first concussion may have led to an incomplete recovery, thereby increasing vulnerability to more severe damage during the subsequent head injury.

The persistence of emotional challenges, even after considerable HBOT and neurofeedback intervention, reveals the intricate challenges associated with TBI recovery. The patient’s favorable response to continued neurofeedback and qEEG monitoring demonstrates that iterative assessments and prolonged neurorehabilitation can be essential for achieving optimal long-term outcomes. Concurrently, ongoing HBOT sessions contributed to symptomatic relief and improved stress management, suggesting a synergistic effect between these therapeutic modalities.

Notably, the patient’s trajectory shows that rehabilitation may extend beyond the acute phase and often calls for repeated, carefully structured interventions. Integrating HBOT, neurofeedback, and targeted medical evaluations, including analyses of neurotransmitter and genetic factors, appears to have generated durable gains in cognitive, emotional, and functional well-being.

Several key themes emerge by situating this patient’s experiences within the broader literature on concussion management: the wide-ranging manifestations of persistent PCS, the current hurdles in defining universally accepted mTBI biomarkers, and the demonstrated value of adopting a multidisciplinary framework. This combined approach may be especially beneficial for individuals who suffer multiple concussions within a short timeframe, underscoring the importance of sustained and multifaceted care.

Persistence and heterogeneity of post-concussion syndrome

Consistent with epidemiological data, mTBI and PCS can manifest following various incidents, including sports injuries, vehicular accidents, and falls [1]. Although most concussions resolve within weeks, approximately 10-15% of individuals experience prolonged symptoms spanning months or years [1,2]. The patient’s gradual development of severe PCS after the second head injury, characterized by profound brain fog, lethargy, reduced appetite, and cognitive impairment, attests to the variability and sometimes insidious progression of concussion sequelae. This heterogeneity underscores mTBI pathophysiology's complexity, wherein structural and functional factors contribute to persistent dysfunction [2,3]. Consequently, clinical practice necessitates a comprehensive, personalized approach that addresses each patient’s constellation of symptoms.

Role of HBOT in mTBI rehabilitation

HBOT has been proposed as a mechanism to facilitate enhanced oxygenation in cerebral tissues, thus potentially aiding in the repair of injured neurons and mitigating secondary injury processes [6,7]. Early studies by Harch et al. [6] have documented promising improvements in cognition, mood, sleep quality, and overall functioning among patients with persistent PCS. Similarly, a systematic review by Harch [7] supported the efficacy of 1.5 atmospheres absolute (ATA) in ameliorating cognitive deficits, with an American Society of Plastic Surgeons Class A Recommendation for treating PCS at this pressure range. However, other reviews and randomized controlled trials have produced conflicting findings, indicating that HBOT’s therapeutic benefit may vary according to dosage (pressure and oxygen concentration), patient selection, and study methodology [8,10-12].

In this case, the patient underwent HBOT repeatedly and in large cumulative volumes, initially 11 sessions after the first concussion, followed by 80 consecutive sessions after the second, and a continuing protocol for maintenance. Notably, the patient ascribed considerable subjective and functional improvement to HBOT, aligning with prior evidence suggesting that specific subgroups may benefit more profoundly [9]. The repeated interventions further highlight the importance of adequate treatment duration; the patient’s decision to discontinue prematurely after the first concussion may have left areas of cerebral dysfunction under-addressed, ultimately exacerbating susceptibility to subsequent injury.

Neurofeedback as a complementary modality

Growing evidence points to neurofeedback as an adjunctive therapy capable of attenuating or remediating neurocognitive deficits following TBI [14-18]. Neurofeedback aims to restore more normalized brainwave patterns and enhance neuroplasticity by providing real-time EEG-based feedback. As presented in multiple controlled studies, neurofeedback interventions have shown promise in improving attention, working memory, and emotional regulation [15-17]. Some investigations report gains in short-term memory or attention indices (e.g., the Continuous Attention Test and the Wechsler Memory Scale), while others, such as Rostami et al. [17], found no statistically significant improvements under specific training protocols.

In the present case, the patient initiated neurofeedback to address persisting brain fog, impaired executive function, and emotional dysregulation. By targeting cortical areas identified through qEEG as dysregulated, neurofeedback sessions led to self-reported mental clarity, decision-making, focus, and emotional stability enhancements. Subsequent qEEG data substantiated the subjective improvements, with objective markers indicating reduced aberrant activity in previously affected regions. Significantly, the patient’s processing speed and irritability improvements after the second wave of neurofeedback in 2024 reinforce existing literature that supports repeated or extended neurofeedback sessions for optimal rehabilitation [15,18].

Synergistic effects and multidisciplinary considerations

The patient’s ongoing recovery, characterized by sustained and progressive improvements, highlights a potential synergistic benefit arising from the combined use of HBOT and neurofeedback. By targeting different yet complementary aspects of brain injury, HBOT addressing issues of cerebral hypometabolism and tissue oxygenation, and neurofeedback focusing on irregular electrical activity and network dysfunction, this dual-modality approach offers promise for enhancing therapeutic outcomes.

Implications for clinical practice and guidelines include building on literature advocating multifaceted interventions for TBI [2,3], the patient’s clinical course underscores the importance of individualized, flexible care. Recommended guidelines include early screening and intervention as well as prompt assessment to identify candidates for HBOT and neurofeedback as soon as symptoms are noted which may optimize recovery potential, as immediate therapy can help prevent secondary complications [2,3]. It is important to use baseline measurements using standardized assessment tools such as qEEG, Neurobehavioral Symptom Inventory (NSI), or Disability Rating Scale (DRS) at the outset to establish objective benchmarks for tracking progress.

An integrated, multidisciplinary approach through team coordination points toward a collaboration among neurologists, rehabilitation specialists, and mental health professionals to ensure that interventions like HBOT and neurofeedback are harmonized with other supportive treatments such as cognitive therapy, lifestyle modifications, and pharmacological management [2,3].

Personalized protocols consisting of adapting the frequency, duration, and specific parameters of HBOT and neurofeedback to individual symptom profiles can enhance efficacy, as demonstrated by this patient’s custom-tailored sessions and iterative clinical evaluations. Also, ongoing monitoring and adjustments and periodic follow-up qEEG scans help clinicians fine-tune neurofeedback protocols according to evolving brainwave patterns [2,3].

Flexible HBOT protocols involve adjusting oxygen pressure and session length based on observed responses, both subjective improvements and objective data, maximizing therapeutic impact while minimizing risks. Clinical emphasis on functional outcomes using real-life metrics allows clinicians to monitor daily functioning, activities of daily living, cognitive tasks, and psychological well-being, to confirm that interventions translate into meaningful benefits outside the clinic. Patient-centered goals involve regularly revisiting and updating treatment objectives with patient input to encourage motivation, adherence, and long-term improvement [2,3].

In terms of addressing potential placebo effects, when combining HBOT and neurofeedback, placebo effects may arise from the patient’s expectations of novel, high-tech therapies. Several measures can mitigate these concerns. Objective measurement tools through the use of validated instruments like the NSI, Disability Rating Scale, and repeated qEEG recordings help distinguish genuine physiological change from patient or clinician bias. Blinded assessments, whenever feasible, incorporate blinded evaluators for certain clinical outcome measures which can reduce expectancy effects [2,3].

Consistency and documentation through detailed logs of session parameters, medication adjustments, and concurrent therapies ensure that any observed changes can be correlated with specific interventions rather than extraneous variables [2,3].

Overall, this patient’s care trajectory, marked by iterative consultations and personalized treatment protocols, aligns with best-practice guidelines recommending early, adaptable, and symptom-focused therapy [3]. It further illustrates that combining HBOT and neurofeedback within a broader rehabilitation framework may yield pronounced and enduring benefits for individuals recovering from TBI. However, larger-scale studies with robust controls are warranted to validate these findings, refine protocols, and substantiate the synergy between these two modalities in routine clinical practice [2,3].

Limitations and future directions

Although the present case details a successful outcome, several limitations should be acknowledged. First, as a single-case report, it is impossible to conclude generalizability. Intrinsic variables could influence treatment responsiveness, such as the patient’s unique neurological baseline, the timing and total volume of HBOT sessions, or his motivation to adhere to neurofeedback. Second, the absence of objective neuroimaging across all phases of treatment, and lack of qEEG results, constrains our ability to delineate neuroanatomical recovery pathways. Also, placebo effects cannot be excluded; the extended course of care and extensive patient-clinician interactions could contribute to perceived improvements.

Given these constraints, more extensive randomized controlled trials are warranted, or simple repeated measures qEEG studies. Future research might evaluate the interplay of precise HBOT pressures (1.5 vs. 2.0 ATA), frequency and duration of neurofeedback sessions, and potential moderators like genetic predispositions or concurrent pharmacotherapies. Additionally, a standardized outcome battery encompassing neuroimaging markers, neuropsychological measures, and quality-of-life indices would yield a clearer understanding of combined or sequential HBOT and neurofeedback interventions for mTBI.

Neurofeedback therapy can be quite expensive due to the specialized equipment and trained professionals required to administer it. Access to neurofeedback therapy can be limited, especially in rural or underserved areas. Insurance may not always cover neurofeedback therapy, or it may only cover a portion of the costs. This can make it financially challenging for many patients to afford the necessary number of sessions.

Also, the authors deliberately refrained from providing extensive qEEG data and imaging results in order to emphasize qualitative clinical processes and quantitative functional outcomes (NSI), rather than to offer definitive diagnostic metrics. Detailed imaging analyses can sometimes overshadow the practical importance of real-world changes, such as symptom alleviation and improvements in daily functioning that fundamentally determine a treatment’s success. In line with this perspective, the authors chose to foreground observable clinical progress and patient-reported improvements, keeping the focus squarely on meaningful, life-enhancing benefits.

## Conclusions

This patient, who experienced two mTBIs over approximately 14 months, achieved marked self-reported clinical and statistically significant improvement through HBOT and neurofeedback interventions. The patient’s case underscores the importance of adhering to recommended HBOT protocols, employing neurofeedback to address persistent cognitive and affective deficits, and integrating ongoing evaluations to adjust therapeutic strategies over time. Maintenance of HBOT further contributed to stress reduction and neurological support. This comprehensive, individualized approach may represent a practical model for other patients with recurrent mTBIs, emphasizing the need for long-term follow-up and integrative therapies to achieve optimal recovery.
